# The activity and functions of soil microbial communities in the Finnish sub-Arctic vary across vegetation types

**DOI:** 10.1093/femsec/fiac079

**Published:** 2022-07-01

**Authors:** Sirja Viitamäki, Igor S Pessi, Anna-Maria Virkkala, Pekka Niittynen, Julia Kemppinen, Eeva Eronen-Rasimus, Miska Luoto, Jenni Hultman

**Affiliations:** Department of Microbiology, 00014 University of Helsinki, Helsinki, Finland; Department of Microbiology, 00014 University of Helsinki, Helsinki, Finland; Helsinki Institute of Sustainability Science (HELSUS), 00014 University of Helsinki, Helsinki, Finland; Department of Geosciences and Geography, 00014 University of Helsinki, Helsinki, Finland; Woodwell Climate Research Center, MA, 02540-1644, USA; Department of Geosciences and Geography, 00014 University of Helsinki, Helsinki, Finland; Geography Research Unit, 90014 University of Oulu, Oulu, Finland; Department of Microbiology, 00014 University of Helsinki, Helsinki, Finland; Marine Research Centre, Finnish Environment Institute (SYKE), 00790, Helsinki, Finland; Helsinki Institute of Sustainability Science (HELSUS), 00014 University of Helsinki, Helsinki, Finland; Department of Geosciences and Geography, 00014 University of Helsinki, Helsinki, Finland; Department of Microbiology, 00014 University of Helsinki, Helsinki, Finland; Helsinki Institute of Sustainability Science (HELSUS), 00014 University of Helsinki, Helsinki, Finland; Soil Ecosystems Group, Natural Resources Institute Finland, 00790 Helsinki, Finland

**Keywords:** climate change, microbial communities, microbial ecology, transcriptomics, tundra

## Abstract

Due to climate change, increased microbial activity in high-latitude soils may lead to higher greenhouse gas (GHG) emissions. However, microbial GHG production and consumption mechanisms in tundra soils are not thoroughly understood. To investigate how the diversity and functional potential of bacterial and archaeal communities vary across vegetation types and soil layers, we analyzed 116 soil metatranscriptomes from 73 sites in the Finnish sub-Arctic. Meadow soils were characterized by higher pH and lower soil organic matter (SOM) and carbon/nitrogen ratio. By contrast, dwarf shrub-dominated ecosystems had higher SOM and lower pH. Although Actinobacteria, Acidobacteria, Alphaproteobacteria and Planctomycetes were dominant in all communities, there were significant differences at the genus level between vegetation types; plant polymer-degrading groups were more active in shrub-dominated soils than in meadows. Given that climate-change scenarios predict the expansion of shrubs at high latitudes, our results indicate that tundra soil microbial communities harbor potential decomposers of increased plant litter, which may affect the rate of carbon turnover in tundra soils. Additionally, transcripts of methanotrophs were detected in the mineral layer of all soils, which may moderate methane fluxes. This study provides new insights into possible shifts in tundra microbial diversity and activity due to climate change.

## Teaser

Active microbial communities were significantly different in the organic and mineral soil layers and the communities differed significantly between four different vegetation types both in the organic and mineral layers.

## Introduction

The Arctic is one of the regions experiencing the most rapid and severe effects of climate change (IPCC 2021). Major ecological disturbances have already been observed in Arctic ecosystems and are expected to become more frequent over the coming decades, even if anthropogenic greenhouse gas (GHG) emissions are curbed (Post et al. 2019). For example, a systematic greening of the Arctic tundra has been observed over recent decades, accompanied by increased plant productivity and northward and upslope expansion of tall shrubs and trees into this otherwise treeless biome (Frost and Epstein 2014, Heijmans et al. 2022). In addition to regional disturbances, the effects of Arctic climate change may have wider global consequences due to the large amounts of carbon (C) and nitrogen (N) stored in frozen tundra soils (Mackelprang et al. 2011, Johnston et al. 2019). Given that warmer temperatures lead to increased rates of soil decomposition and GHG release, Arctic organic matter stocks can contribute to a positive warming feedback loop (Bond-Lamberty and Thomson 2010, Jansson and Hofmockel 2020).

Microorganisms are important drivers of nutrient cycling in the tundra, and thus the investigation of how microbial communities respond to local environmental variation in tundra soils is essential to predict the impacts of climate change on the GHG budget of this biome (Buckeridge et al. 2013, Virkkala et al. 2018, Mod et al. 2021). Tundra microbial communities are shaped by extreme environmental stressors, such as fluctuating temperatures, long periods of sub-zero temperatures, frequent freeze-thaw events, intensive UV radiation and drought. However, microbial community composition is stable and diverse across seasons in the sub-Arctic tundra, with Acidobacteria being the predominant phylum in acidic soils (Pessi et al. 2022a, Männistö et al. 2007, 2013). Soil microbes participate in organic matter decomposition, methanogenesis and methanotrophy in the high-Arctic and permafrost soil ecosystems (e.g. Hultman et al. 2015, Schostag et al. 2015, Tveit et al. 2015), including in peatlands that experience permafrost thaw (McCalley et al. 2014, Singleton et al. 2018, Woodcroft et al. 2018). However, their functional potential has not been explored in detail due to technical limitations and their vast diversity. This, in turn, precludes a comprehensive understanding of the contribution of tundra microorganisms to and their feedback with climate change. A better understanding of tundra microbial communities and their functions is key to acquiring process-level knowledge on the biogeochemistry of and to develop accurate models of GHG cycling.

At large geographic scales, the composition of Arctic tundra vegetation is primarily shaped by climate (e.g. mean summer temperature) (Walker et al. 2005). However, tundra vegetation is typically heterogeneous at the local level, as growing conditions (microclimate, soil moisture, soil nutrients) vary greatly at small spatial scales (Kemppinen et al. 2021a, le Roux et al. 2013). Different vegetation types affect soil biotic and abiotic factors, which, in turn, influence the local soil microbial community. For example, tundra soils have relatively low pH (4–6) (Hobbie and Gough 2002, Männistö et al. 2007), which is generally one of the most important drivers of microbial community composition and activity (Chu et al. 2010). In addition, the quantity and quality of soil organic matter (SOM), N availability and C/N ratio affect microbial processes and primary production in tundra soils (Koyama et al. 2014, Zhang et al. 2014). Despite a great local heterogeneity, only fragmentary knowledge exists regarding microbial community composition and activity across different vegetation types in the drier upland tundra, which is a noteworthy ecosystem that covers approximately 90% of the Arctic (Walker et al. 2005). This knowledge gap is relevant as most of the Arctic is greening and the typically low-growing Arctic vegetation is being gradually replaced by taller woody plants, a development known as shrubification (Mod and Luoto 2016, Myers-Smith et al. 2020, Heijmans et al. 2022). Shifts in vegetation and, in particular, shrub expansion across the Arctic tundra are some of the most important ecosystem responses to climate change. These shifts in vegetation potentially alter ecosystem carbon balances by affecting a complex set of soil-plant-atmosphere interactions (Mekonnen et al. 2021). In general, decomposition in the Arctic is slower than plant growth, causing a build-up of detritus in tundra soils. Climate warming may result in carbon loss by accelerating the decomposition of SOM. Future C storage in the Arctic tundra will depend on the balance of C losses from SOM and C storage in plant pools due to higher productivity and changes in plant community assemblages (Weintraub and Schimel 2005).

Although the warming trend in the Arctic region is alarming, the response of Arctic ecosystems to climate change is only poorly understood. The effects of climate change are particularly complex in tundra ecosystems, given their high biotic and abiotic heterogeneity. Thus, elucidating the taxonomic and functional composition of microbial communities across tundra soils is essential for a better understanding of the effects of climate change on the Arctic and potential feedbacks with the global climate system. Here, we used metatranscriptomics to investigate the activity of microbial communities during the active growing season across 73 sites in a mountain tundra ecosystem in the Finnish sub-Arctic, covering different vegetation types and a wide range of microclimatic and soil nutrient conditions. We sought to investigate the effect of different vegetation types and soil nutrient conditions on microbial diversity and activity in tundra soils to obtain insights into potential future changes on microbial communities and functions associated with the increased greening of Arctic ecosystems.

## Materials and methods

### Study setting and sampling

**Figure 1.**
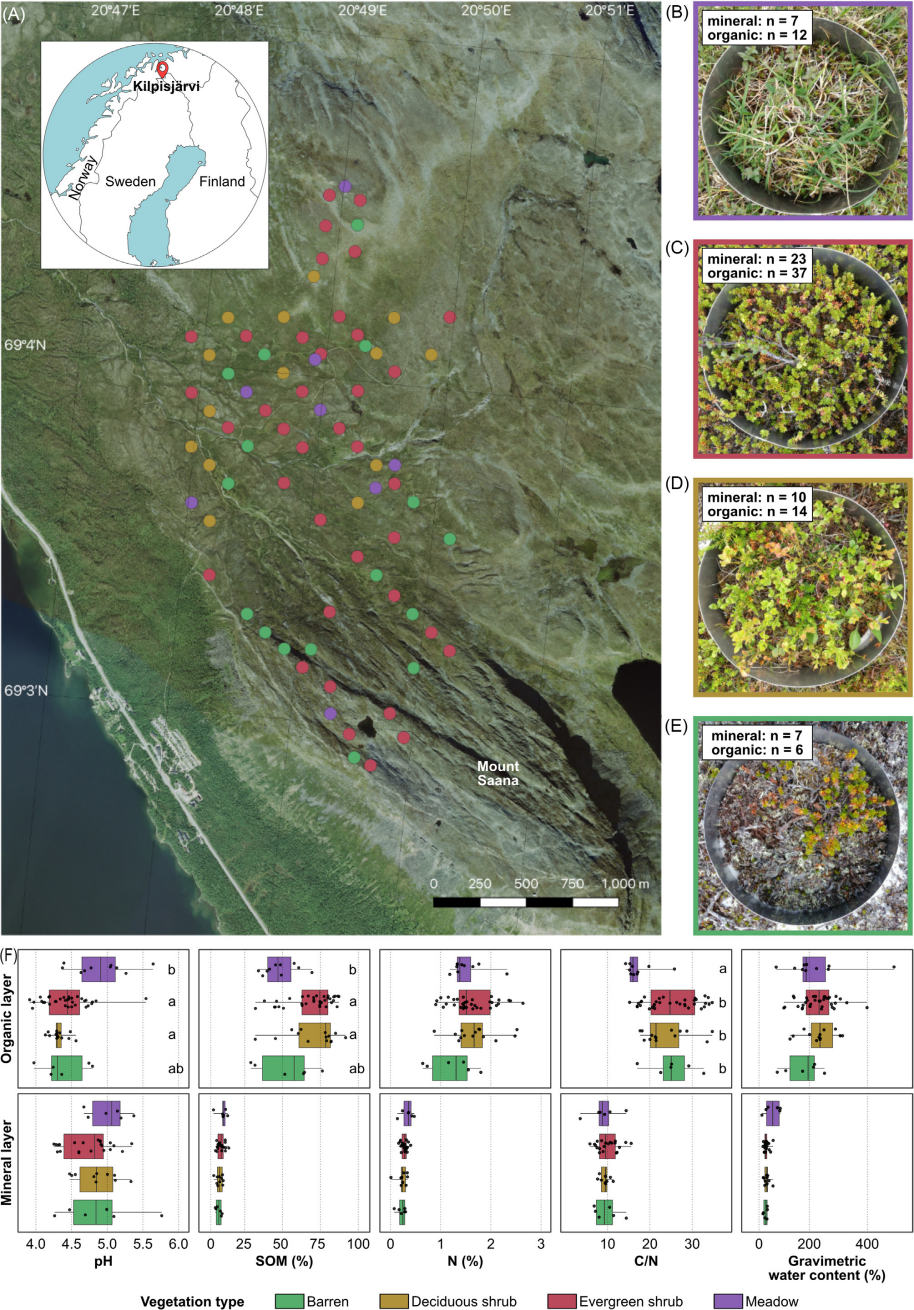
**(A)** Map of the sampling sites in Kilpisjärvi, northwestern Finland. The inset shows the location of Kilpisjärvi in Fennoscandia. **(B–E)** Pictures of the four types of soil vegetation studied (B: meadow; C: evergreen shrub; D: deciduous shrub, E: barren). The number of samples analyzed from each vegetation type is indicated. **(F)** Soil physicochemical properties across the four vegetation types. Categories with the same letter are not statistically different (one-way ANOVA, *P* > 0.05). One outlier was removed from the mineral C/N plot.

The study area was located in Kilpisjärvi, northwestern Finland, and extends to the Scandinavian Mountains (Fig. 1). The 3-km^2^ area covers parts of two mountains, Mount Saana (69°02′N, 20°51′E) and Mount Korkea-Jehkas (69°04′N, 20°79′E), and the valley between. The elevation range in the study area is 320 m, with the highest point on Mount Saana at 903 m a.s.l. The study area is topographically heterogeneous and is part of the sub-Arctic alpine tundra biome. Consequently, the area contains relatively broad environmental gradients of soil microclimate, moisture and pH, among others (Kemppinen et al. 2021a). The vegetation type is mainly mountain heath dominated by dwarf shrubs (such as *Empetrum nigrum* and *Betula nana*) and to a lesser extent by *Juniperus communis, Vaccinium vitis-idaea, V. uliginosum* and *V. myrtillus* (Kemppinen et al. 2019). However, due to fine-scale environmental variation and broad gradients, the landscape forms a mosaic of different vegetation types, as both vegetation cover and plant-species composition can vary over very short distances (le Roux et al. 2013). The soils in the area are mostly poorly developed leptosols with shallow organic layers and occasional podzolization; however, the meadows have soils with thicker organic layers. Permafrost is absent from these soils but can be found in the bedrock above 800 m a.s.l. (King and Seppälä 1987). The average air temperature and precipitation in July for the period 1981–2010 measured at the Kilpisjärvi meteorological station (69°05′N 20°79′E, 480  m a.s.l.) were 11.2°C and 73 mm, respectively (Pirinen et al. 2012).

Samples were collected in July 2017 from 73 sites (Fig. 1a, Supplementary Table 1). Sampling sites encompassed four vegetation types, namely barren soil, deciduous shrub, evergreen shrub and meadow (Fig. 1b-e), which were classified according to the Circumpolar Arctic Vegetation Map (Walker et al. 2005). All sampling equipment was disinfected with 70% ethanol before and between samples to avoid contamination. The soil was bored with a 50-mm diameter stainless-steel soil corer with a plastic inner casing. When available, both organic and mineral layer sub-samples were collected. Sampling was targeted below the plant roots, with a 5-cm target depth for the organic layer sample. The mineral layer sample was taken from the lowest part of the core, from 10 to 15 cm. The samples were placed in a Whirl-Pak sampling bag (Nasco, Fort Atkinson, WI, USA) with a metal spoon and immediately frozen on dry ice and kept frozen at ^–^80°C until nucleic acid extraction. Samples were collected from 73 sites, from which 116 metatranscriptomes were sequenced.

### Soil physicochemical data

For the analysis of soil physicochemical properties, approximately 0.2 dm^3^ of soil was collected with a steel cylinder and stored at 4°C. The soils were lyophilized according to the Finnish standard SFS300 and pH was analyzed according to international standard ISO10390. SOM content was determined by loss on ignition analysis according to the Finnish standard SFS3008. CNS analysis (carbon, nitrogen and sulfur) was performed with a Vario Micro Cube analyzer (Elementar, Langenselbold, Germany). For this, mineral samples were sieved through a 2-mm plastic sieve and the organic samples were homogenized by hammering the material into smaller pieces. Differences in soil physicochemical properties between vegetation types were assessed using the Kruskal-Wallis test followed by pairwise Wilcoxon test with Bonferroni correction (functions *kruskal.test* and *pairwise.wilcox.test*, R-core package).

### Nucleic acid extraction

Three replicate nucleic acid extractions were performed for each sample. Samples were kept on ice during weighing and extraction and all steps were performed promptly with nuclease-free labware to avoid RNA degradation. Solutions and water were treated with 0.1% diethylpyrocarbonate. Nucleic acids were extracted using a modified hexadecyltrimethylammonium bromide (CTAB), phenol-chloroform and bead-beating protocol (Griffiths et al. 2000, DeAngelis et al. 2009). On dry ice, approximately 0.5 g of frozen soil was transferred to a 2-ml Lysing Matrix E tube (MP Biomedicals, Heidelberg, Germany) and 0.5 ml of CTAB buffer (consisting of equal amounts of 10% CTAB in 1 M sodium chloride and 0.5 M phosphate buffer in 1 M NaCl), 50 μl of 0.1 M ammonium aluminum sulfate (NH_4_(SO_4_)_2_ ·12 H_2_O) and 0.5 ml phenol: chloroform: isoamyl alcohol (25 : 24 : 1) was added. After bead-beating with FastPrep (MP Biomedicals, Heidelberg, Germany) at 5.5 m s^–1^ for 30 s, 0.5 ml of chloroform was added. Nucleic acids were precipitated with polyethylene glycol 6000 (PEG6000, 30% in 1.6 M NaCl) and washed with ethanol. Nucleic acids were extracted again from the leftover soil pellet to maximize yields. Nucleic acids were resuspended in 25 μl of Buffer EB and 250 μl of Buffer RLT with β-mercaptoethanol was added. Buffers EB and RLT were from an AllPrep DNA/RNA Mini Kit (Qiagen, Hilden, Germany). All centrifugations were performed at 4°C. RNA and DNA were purified with AllPrep DNA/RNA Mini Kit (Qiagen, Hilden, Germany), where RNA was treated with DNAse I. The amount and integrity of RNA were assessed on a Bioanalyzer RNA 2100 Nano/Pico Chip with Total RNA Assay (Agilent, Santa Clara, CA, USA). To ensure that RNA was DNA-free, PCR with universal primers and gel electrophoresis was performed. Triplicates were pooled by combining equal amounts of RNA from each triplicate. Ribosomal RNA was not depleted and thus the total RNA approach was used (Urich et al. 2008).

### Sequencing

Complementary DNA (cDNA) libraries were constructed with the Ultra II RNA Library Prep Kit for Illumina (New England Biolabs, Ipswich, MA, USA). cDNA concentrations were measured using a Qubit fluorometer with a dsDNA BR/HS kit (Invitrogen, Carlsbad, CA, USA). Before sequencing, the libraries were analyzed with Fragment analyzer (Advanced Analytical, Ames, IA, USA) and small cDNA fragments were removed to avoid primer binding to the flow cell and to reduce cluster density. Single-end sequencing was performed on an Illumina NextSeq 500 (Illumina, San Diego, CA, USA) with 150 cycles at the Institute of Biotechnology, University of Helsinki, Finland.

### Metatranscriptomic data processing and analysis

One sample (site 11 202, organic layer) yielded a much higher than average amount of reads (80.4 million) and was randomly reduced to 4 million reads with seqtk v. 1.3 (https://github.com/lh3/seqtk). Sequence quality was assessed with FastQC v. 0.11.5 (https://www.bioinformatics.babraham.ac.uk/projects/fastqc) and MultiQC v. 1.3 (Ewels et al. 2016). Trimming and quality filtering were performed with Cutadapt v. 1.10 (Martin 2011), applying a quality cut-off of 25 and a minimum adapter overlap of 10 bp. Metaxa2 v. 2.1.3 (Bengtsson-Palme et al. 2015) was used to identify reads mapping to the small subunit (SSU) rRNA. These were then classified against the SILVA database release 132 (Quast et al. 2013) using the mothur v. 1.40.5 classify.seqs command with a confidence cut-off of 60% (Schloss et al. 2009). The taxonomy of abundant taxa was manually updated according to the Genome Taxonomy database (Parks et al. 2018, 2020). For analysis of protein-coding genes, reads were mapped to the Kyoto Encyclopedia of Genes and Genomes (KEGG) Prokaryote database release 86 (Kanehisa and Goto 2000) using DIAMOND blastx v. 2.1.3 (Buchfink et al. 2015) with an E-value cut-off of 0.001. The KEGG orthology (KO) identifier of the best hit was assigned to each read and mapped to the KEGG module hierarchy, and spurious pathways were removed using MinPath v. 1.4 (Ye and Doak 2009). Because genes for methane and ammonia oxidation (*pmo* and *amo*, respectively) are not distinguished in KEGG, we used blastx (Altschul et al. 1990) to compare putative *pmoA*-*amoA* genes against a manually curated database of five PmoA and nine AmoA sequences from both bacteria and archaea (Supplementary Fig. 1).

Statistical analyses and visualization were performed with R v. 3.6.2 (R Core Team 2020). For multivariate analyses, taxonomic (genera abundances) and functional (KO abundances) matrices were transformed into Bray-Curtis distance matrices (function *vegdist*, R-package vegan v. 2.5–6; https://github.com/vegandevs/vegan). Community-wide differences between vegetation types and soil layers were tested with permutational analysis of variance (PERMANOVA) (function *adonis*, R-package vegan v. 2.5–6) followed by pairwise PERMANOVA (function pairwise.perm.manova, R-package RVAideMemoire v.0.9–78; https://cran.r-project.org/web/packages/RVAideMemoire). Differences in community structure were visualized using principal coordinates analysis (PCoA) (function *ordinate*, R-package Phyloseq v. 1.30.0; McMurdie and Holmes 2013). The relationship between community structure and soil physicochemical properties was assessed using distance-based redundancy analysis (db-RDA) with forward selection (functions *capscale* and *ordistep*, R-package vegan v. 2.5–6). Soil physicochemical data were log-transformed prior to analysis. Differences in the abundance of individual bacterial and archaeal genera and functional genes between vegetation types were tested with one-way analysis of variance (ANOVA) (functions *lm* and *aov*, R-core package) followed by pairwise *t*-test (function *pairwise.t.test*, R-core package).

### Data availability

Sequences were deposited in the European Nucleotide Archive under accession number PRJEB45463.

## Results

### Soil properties across vegetation types

We observed high variability in soil properties between samples from the organic and mineral layers and across vegetation types. SOM content varied from 2% to 92%, gravimetric soil water concentration from 9% to 432% and pH from 3.9 to 5.8 (Fig. 1f). Soil pH was generally higher in the mineral layer, whereas SOM, N, C/N ratio and gravimetric water content were higher in the organic layer. Soil properties also differed between vegetation types (Kruskal-Wallis test; *P* < 0.01). In the organic layer, meadow sites were less acidic and contained less SOM than sites dominated by deciduous or evergreen shrubs. Meadows also had a lower C/N ratio than shrubs or barren soils. The physicochemical properties of the mineral layer did not differ significantly between vegetation types. Varying degrees of collinearity between the physicochemical variables was observed (Supplementary Fig. 2).

### Microbial community composition along the tundra landscape

We obtained 281.1 million sequence reads using a total RNA metatranscriptomic approach (Urich et al. 2008). First, we analyzed reads corresponding to the small subunit ribosomal RNA (SSU rRNA). SSU rRNA represented 35 ± 2% (mean ± standard deviation) of the reads. Over 80% of the SSU rRNA sequences were bacterial, 0.1% archaeal (mostly Thaumarchaea) and 19% were of eukaryotic origin, with fungal SSU rRNA representing 12% of the sequences (Ascomycota, 8%; Basidiomycota, 3%). Furthermore, 0.2% of the sequences were recognized as SSU rRNA but could not be assigned unambiguously to a specific domain.

**Figure 2.**
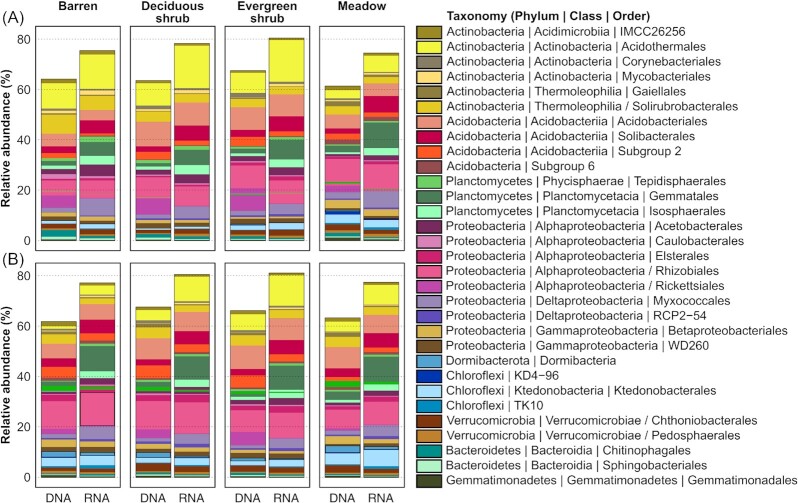
Relative abundances of bacterial orders in metatranscriptomes (RNA) and metagenomes (DNA) of samples from the **(A)** organic and **(B)** mineral layer. Samples from the same vegetation type were pooled and unclassified taxa were removed.

**Figure 3.**
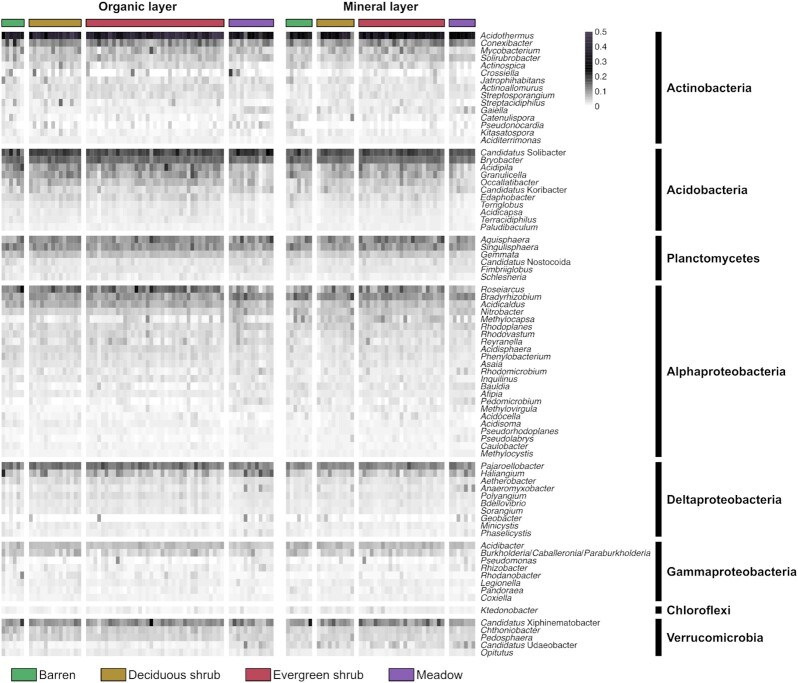
Relative abundances of the 80 most abundant bacterial genera across the samples. Abundances were square root-transformed to improve visualization.

The predominant bacterial groups were assigned to the phylum Actinobacteria (27 ± 9% of the sequences; orders Acidothermales and Solirubrobacterales), phylum Acidobacteria (17 ± 3%; orders Acidobacteriales and Solibacterales), class Alphaproteobacteria (16 ± 3%; orders Rhizobiales and Acetobacterales) and phylum Planctomycetes (14 ± 3%; order Gemmatales) (Fig. 2). Classes Deltaproteobacteria and Gammaproteobacteria and phyla Chloroflexi and Verrucomicrobia were also abundant. The most abundant genera were *Acidothermus* (phylum Actinobacteria; 13 ± 5.7%) and *Ca*. Solibacter (phylum Acidobacteria; 3.11 ± 0.83%) followed by *Bryobacter* (phylum Acidobacteria; 2.06 ± 0.38%), *Pajaroellobacter* (class Deltaproteobacteria; 1.64 ± 0.55%) and *Roseiarcus* (class Alphaproteobacteria; 1.48 ± 1.07%) (Fig. 3, Supplementary Table 2).

When comparing SSU rRNA sequences from metatranscriptomes (i.e. RNA, representing active microbes) with sequences from metagenomes (i.e. DNA, representing the whole microbial community) (Pessi et al. 2022a), the most abundant microbial groups were largely the same but with notable differences in abundances (Fig. 2, Supplementary Fig. 3a). Orders with greater relative abundance in the metatranscriptomes included Gemmatales (Planctomycetes), Acidothermales (Actinobacteria), Solibacterales (Acidobacteria) and Myxococcales (Deltaproteobacteria), whereas Subgroup 2 (Acidobacteria), Rickettsiales (Alphaproteobacteria), Dormibacterota, Chitinophagales (Verrucomicrobia) and Gemmatimonadales (Gemmatimonadetes) were more abundant in the metagenomes. In the metatranscriptomes, orders with >1% relative abundance accounted together for 78% of the communities compared with 64% in the metagenomes. Additionally, more than 1500 bacterial and archaeal genera were identified from the metatranscriptomes and less than 500 bacterial and archaeal genera from the metagenomes.

### Differences in microbial community composition across vegetation types

Genus-level community structure was significantly different in the organic and mineral layers (PERMANOVA; *R^2^* = 0.07; *P* < 0.001) (Supplementary Fig. 3b). Interestingly, communities also differed significantly across the four different vegetation types both in the organic (*R^2^* = 0.16; *P* < 0.001) and mineral layers (*R^2^* = 0.13; *P* < 0.01). Pairwise analyses revealed that the communities in the organic layer of meadow sites were significantly different from all other vegetation types, whereas communities from the mineral layer differed only between the meadow and evergreen shrub sites (Supplementary Table 3).

**Figure 4.**
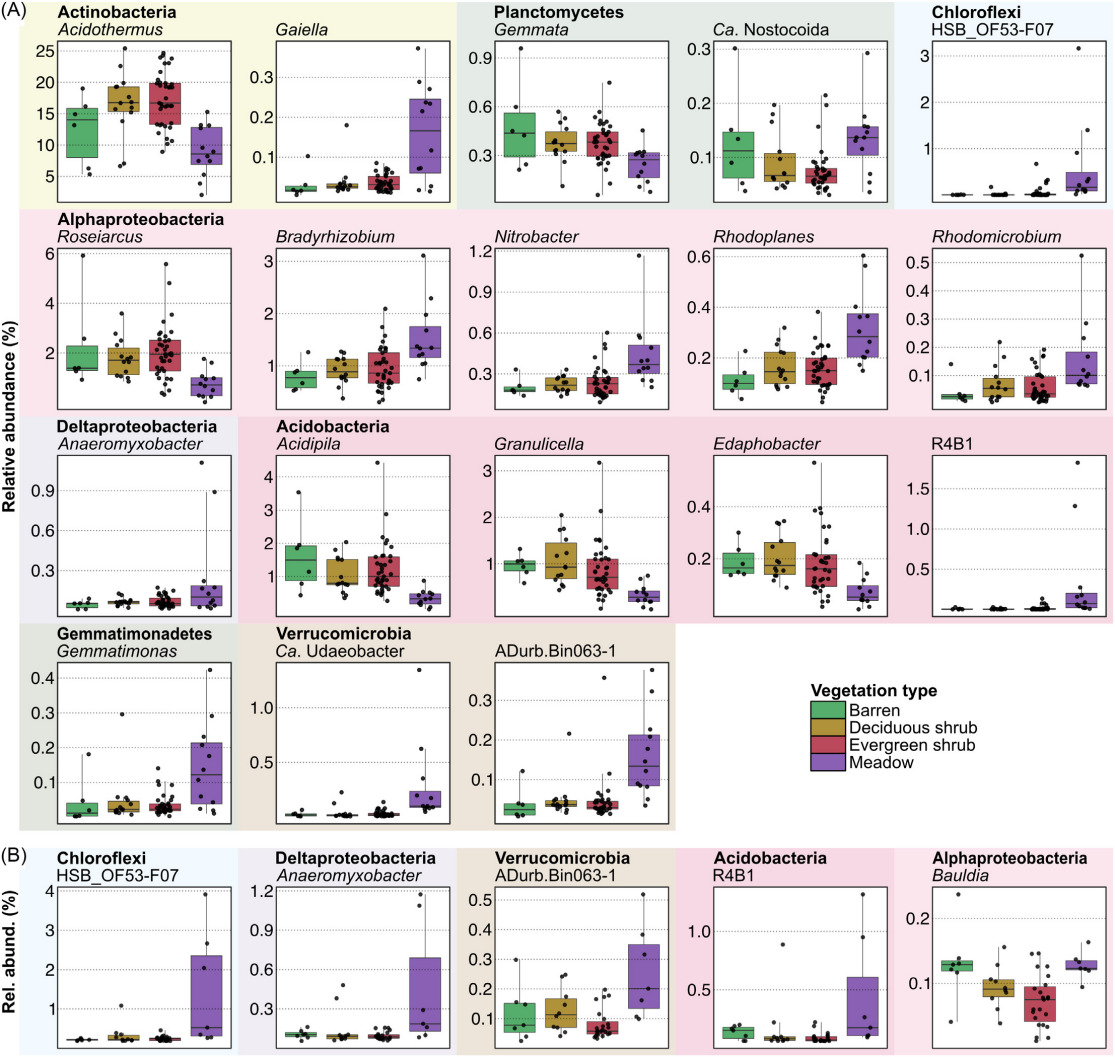
Boxplots showing abundant genera (mean abundance larger than the 2-fold mean of all genera) that were differentially active across vegetation types in **(A)** organic layer and (**B)** mineral layer (one-way ANOVA, *P* < 0.01).

Genus-level comparisons were conducted for the metatranscriptomes across vegetation types and soil layers. For this, we considered only abundant genera (i.e. genera with a mean abundance at least twice the mean of all genera). In the organic layer, the alphaproteobacterial genera *Bradyrhizobium, Nitrobacter, Rhodoplanes* and *Rhodomicrobium* were significantly more abundant in samples from the meadow sites than sites with other vegetation types (ANOVA; *P* < 0.01) (Fig. 4a). The same was observed for other taxa, namely *Gaiella* (Actinobacteria), *Ca*. Nostocoida (Planctomycetes), ‘HSB_OF53-F07’ (Chloroflexi), *Anaeromyxobacter* (Deltaproteobacteria), *Gemmatimonas* (Gemmatimonadetes), ‘R4B1’ (Acidobacteria), *Ca*. Udaeobacter and ‘ADurb.Bin063-1′ (Verrucomicrobia). On the other hand, *Acidothermus* (Actinobacteria) was less abundant in the meadow than the shrub sites, whereas *Roseiarcus* (Alphaproteobacteria), the acidobacterial genera *Acidipila, Granulicella* and *Edaphobacter*, and *Gemmata* (Planctomycetes), were less abundant in the meadow sites than in all other vegetation types. In the mineral layer, ‘HSB_OF53-F07’ (Chloroflexi), *Anaeromyxobacter* (Deltaproteobacteria), ‘Adurb.Bin063-1′ (Verrucomicrobia) and ‘R4B1’ (Acidobacteria) were more abundant in the meadows than all other vegetation types, whereas *Bauldia* (Alphaproteobacteria) was more abundant in the meadows only in relation to the shrub sites (Fig. 4b).

We used db-RDA with forward selection to investigate what factors underlie the observed differences in microbial community structure. In the mineral layer, the best model included vegetation, pH and gravimetric water content (*R^2^* = 0.25; *P* < 0.05), whereas in the organic layer the best model included vegetation, pH and C/N ratio (*R^2^* = 0.28; *P* < 0.05). However, it is important to note that there are varying degrees of collinearity between the variables selected by the forward selection procedure and other variables (Supplementary Fig. 2). For example, gravimetric water content in the mineral layer and pH and C/N ratio in the organic layer correlated with SOM, C and N content (–0.5 ≤ *r* ≥ 0.5). Thus, the variables selected by the model in these cases should be considered, to some extent, as a proxy of the intercorrelated variables.

### Microbial community functions across vegetation types

Differences in protein-coding gene composition between samples from the organic and mineral layers were small (PERMANOVA; *R^2^* = 0.03; *P* < 0.001). Community structure based on protein-coding genes was also significantly different between vegetation types in the organic layer (*R^2^* = 0.10; *P* < 0.001), with communities from the meadow sites differing from shrub and barren sites and evergreen shrub communities differing from barren sites. No significant differences were observed between vegetation types in the mineral layer.

Genes with a KEGG classification represented only a small fraction (1.39 ± 0.27%) of the protein-coding genes. While genes with no KEGG classification were not analyzed in this study, they corresponded mostly to genes encoding proteins with unknown function (Supplementary Fig. 4). The most abundantly transcribed genes that were mapped to KEGG pathways are involved in genetic information processing, including (i) folding, sorting and degradation, (ii) transcription and (iii) metabolism (Supplementary Fig. 5). ABC transporter genes were widely transcribed, including genes encoding transport system substrate-binding proteins for ribose (*rbsB*), D-xylose (*xylF*) and sorbitol/mannitol (*smoE, mtlE*) and multiple sugar transport system ATP-binding proteins (*msmX, msmK, malK, sugC, ggtA, msiK*), indicating the decomposition of plant polymers. Other widely transcribed ABC transporters included genes for branched-chain amino acid transport system proteins (*livKGFHM*) and urea transport system substrate-binding proteins (*urtA*). In addition to transporters, the chaperone genes *groeL* and *dnaK* and the cold-shock protein gene *cspA* (involved in survival in cold temperatures) were among the most transcribed genes across all samples. The gene *coxL*/*cutL* (encoding the large subunit of the aerobic carbon-monoxide dehydrogenase enzyme) and two genes involved in nitrogen uptake (glutamine synthetase [*glnA*] and ammonium transporter [*amt*]) were also widely expressed.

**Figure 5.**
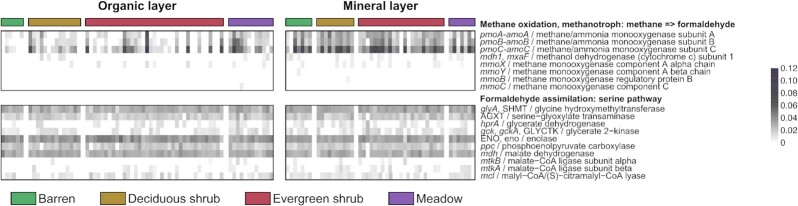
Relative abundances of genes involved in methane oxidation and the serine pathway of formaldehyde assimilation. Abundances were square root-transformed to improve visualization.

Given the high abundance of genes involved in carbohydrate transport, we expanded our analysis to other genes related to C cycling and metabolism (Supplementary Fig. 6). Interestingly, the *pmoABC-amoABC* genes involved in methane/ammonia oxidation were significantly more transcribed in the mineral layer than in the organic layer (ANOVA*; pmoA*: *R^2^* = 0.13, *pmoB*: *R^2^* = 0.09, *pmoC*: *R^2^* = 0.12; *P* < 0.001) (Fig. 5). To further distinguish between the closely related *amoA* and *pmoA* genes, we performed a blastx analysis against a manually curated database of PmoA and AmoA sequences from different organisms. This indicated that 96% of the sequences identified as *pmoA*-*amoA* using the KEGG database correspond to the *pmoA* gene, indicating methane oxidation by the particulate methane monooxygenase (pMMO). However, the soluble methane monooxygenase (sMMO) genes *mmoXYZBCD* and the genes *mxaF* or *xoxF* (encoding methanol dehydrogenases for methanol oxidation to formaldehyde) were generally not transcribed (Fig. 5). Genes for formaldehyde assimilation using the serine pathway, including *glyA* (encoding the enzyme glycine hydroxymethyltransferase), were transcribed. This indicates the utilization of the serine cycle instead of the ribulose monophosphate (*RuMP*) cycle for formaldehyde assimilation in these microbial communities.

## Discussion

We analyzed over 100 soil metatranscriptomes across a sub-Arctic tundra landscape to investigate how microbial community composition and their functions vary across soil layers and vegetation types. Soil physicochemical composition varied according to vegetation type in the organic layer, but not in the mineral layer. This is likely related to vegetation being the primary source of material for the organic layer, whereas the properties of the mineral layer are more affected by bedrock and soil texture, among other factors (Jenny 1941, Haichar et al. 2008). Thus, environmental conditions are more homogeneous in the mineral layer, leading to more uniform microbial communities irrespective of vegetation cover, as observed in the present study. Differences in soil properties between vegetation types were more pronounced in the organic layer, with meadows differing significantly from the other vegetation types by higher pH and lower SOM and C/N ratio. As revealed by our multivariate analyses, these factors were significantly associated with differences in community structure observed between vegetation types, which can be presumably linked to differences in SOM quality and quantity. Actinobacteria, Acidobacteria, Alphaproteobacteria and Planctomycetes were the most active phyla in all vegetation types, which is consistent with previous studies from Arctic regions (Männistö et al. 2007, Hultman et al. 2015, Ivanova et al. 2018, Taş et al. 2018, Tripathi et al. 2019). These phyla, excluding Planctomycetes, were also among the most abundant in the metagenomics dataset from these samples (Pessi et al. 2022a). Archaea, which represented only 0.1% of the transcripts, consisted mostly of Thaumarchaea in the mineral layer as previously observed (Pessi et al. 2022b, Lu et al. 2017, Shao et al. 2019). Communities in both the organic and mineral layers and across all vegetation types were dominated by aerobic acidophilic genera that play a role in the degradation of plant organic matter, including *Acidothermus, Ca*. Solibacter and *Bryobacter* (Mohagheghi et al. 1986, Ward et al. 2009, Kulichevskaya et al. 2010). *Acidothermus* was the most abundant active genus overall and was significantly more abundant in the shrub sites. *Acidothermus cellulolyticus*, the only described species in this genus, is a thermophilic, acidophilic and cellulolytic species first isolated from an acidic hot spring (Mohagheghi et al. 1986). Interestingly, genome analysis of *Ca*. Solibacter revealed not only the ability to utilize complex plant cell-wall polysaccharides and simple sugars but also carbon monoxide (CO), a toxic gas, as a complementary energy source in a mixotrophic lifestyle (Ward et al. 2009). Indeed, the *coxL*/*cutL* gene encoding the carbon monoxide dehydrogenase enzyme responsible for the oxidation of CO was widely expressed in the present study. Furthermore, these microorganisms tolerate temperature and moisture fluctuation and low-nutrient conditions, which are characteristic of tundra soils (Ward et al. 2009, Rawat et al. 2012). In general, our results show that the dominant active microorganisms in the tundra soils studied here are versatile degraders of plant polymers with the ability to thrive in fluctuating conditions and have potential roles in the C cycle.

**Figure 6.**
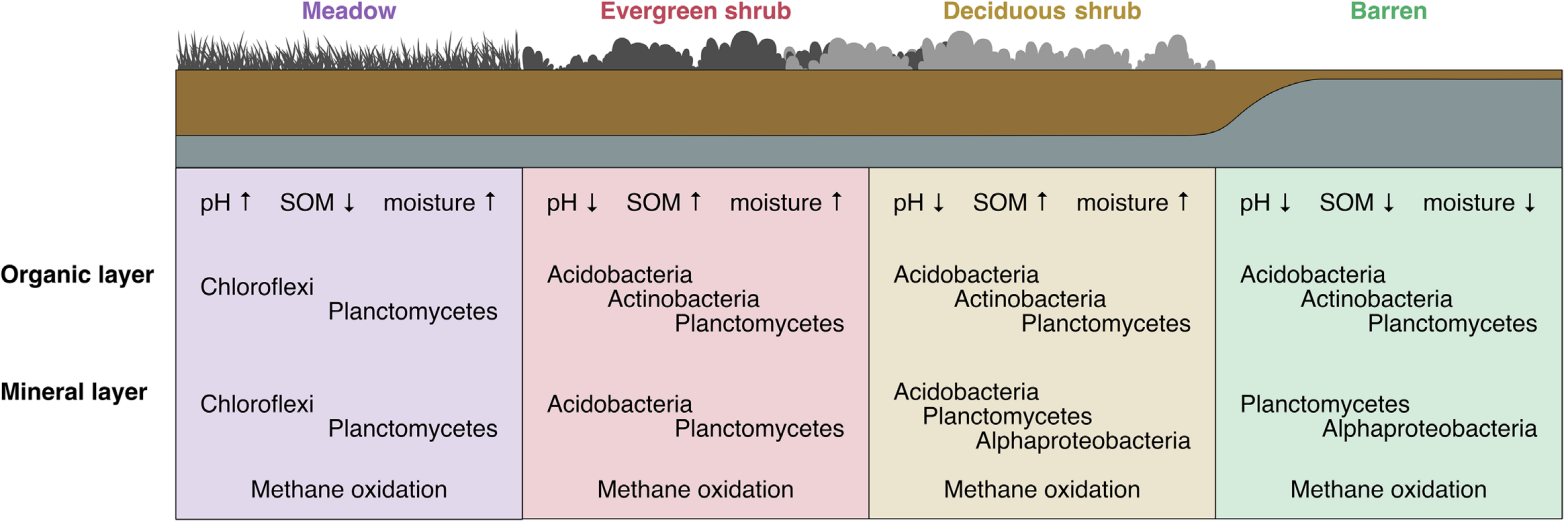
A conceptual figure on the implications of the study. The arrows denote the change in the measured environmental variables and the microbial phyla active in the sites are marked to each vegetation type and soil layer.

Our results provide evidence for a link between soil microbial community composition/activity and vegetation via physicochemical factors, such as pH and SOM content. Meadow soils, which were characterized by higher pH and lower SOM content and C/N ratio, harbored distinct microbial communities compared with the other vegetation types (Fig. 6). Meadows are dominated by forbs, grasses and sedges, which produce litter that decomposes faster and has higher nutrient concentrations with a lower C/N ratio (Hobbie et al. 2000, Eskelinen et al. 2009). Most of the genera that were abundant in the meadows are poorly known, and more research is required to understand their roles in this ecosystem. Interestingly, members of *Gaiella, Bradyrhizobium, Ca*. Udaeobacter and *Gemmatimonas* have been implicated in the cycling of the atmospheric gases H_2_, CO_2_ and N_2_O (Lepo et al. 1980, Park et al. 2017, Severino et al. 2019, Willms et al. 2020). Shrub soils, characterized by lower pH and higher SOM content, had a higher abundance of the Acidobacteria genera *Acidipila, Granulicella* and *Edaphobacter*. These genera most likely have a role in degrading plant-derived organic matter in these shrub soils, as seen in other acidic upland soils (Pankratov and Dedysh 2010, Männistö et al. 2013, Ivanova et al. 2020, 2021). Evergeen shrubs such as *Empetrum nigrum*, which is the dominant plant species in the study area, produce recalcitrant, acidic and relatively slowly decomposing litter. Altogether, our results indicate that shrub soils have a higher abundance of chemoorganotrophs that degrade complex plant polymers, whereas meadows also harbor microbial groups that are not solely dependent on plant-derived organic matter.

Interestingly, our results suggested a potential for methane oxidation in the mineral layer, as the *pmo* gene and genes for the serine pathway were transcribed together with the activity of methanotrophic bacteria such as *Methylocapsa*. Of the known *Methylocapsa* species, *M. gorgona* grows only on methane, whereas *M. acidiphila* and *M. palsarum* also grow on low methanol concentrations and *M. aurea* on methanol and acetate (Dedysh et al. 2002, Dunfield et al. 2010, 2015, Tveit et al. 2019). A recent *in situ*^13^CH_4_-DNA-stable isotope probing (SIP) enrichment study showed that *Methylocapsa* were the dominant active methane oxidizers in high Arctic soil (Altshuler et al. 2022). Based on the results shown here and by others (e.g. Belova et al. 2020), *Methylocapsa* may have a significant role as methane oxidizers in sub-Arctic tundra soils. Moreover, our results showed similar activity levels of methane oxidizers in the mineral layer across all vegetation types, which suggests that methane oxidation is not dependent on vegetation cover in deeper soil layers. Future studies employing e.g. SIP could shed light on the regulation of methane oxidation in tundra soils.

In this study, we investigated the microbial activity across different vegetation types (barren, deciduous shrub, evergreen shrub and meadow) in tundra soils to understand how future changes in vegetation cover (such as shrubification, i.e. decreasing of bare ground and increase in shrub abundance and canopy size) may affect microbial community diversity and activity. For example, in the dwarf shrub-dominated tundra, shrubs influence microclimate, including soil moisture and near ground temperature (Kemppinen et al. 2021b). Although litter abundance is increasing in cold biomes due to shrubification, the increase is not strongly correlated with warming, which may be due to warming-accelerated decomposition (Cornelissen et al. 2007, Elmendorf et al. 2012). Despite the overall similarity in bacterial community composition in this study, shrubs had a more abundant and active community of potential degraders of plant-derived organic matter than other vegetation types. Therefore, we hypothesize that with shrub soils becoming more prevalent, heterotrophic microbial activity may increase and further affect carbon cycling in tundra soils.

## Funding

The study was funded by the Academy of Finland (Grant no. 1314114) and the University of Helsinki's three-year grant to JH. SV was funded by the Doctoral Program in Microbiology and Biotechnology (MBDP). PN was funded by Kone Foundation and Nessling Foundation. JK was funded by the Arctic Interactions at the University of Oulu and Academy of Finland (Grant no. 318930, Profi 4). AMV acknowledges the Gordon and Betty Moore Foundation (Grant 8414).

## Supplementary Material

fiac079_Supplemental_Files
