# Supplementary material for: The activity and functions of soil microbial communities in the Finnish sub-Arctic vary across vegetation types
Source: FEMS Microbiol Ecol. 2022 Jul 1;98(8):fiac079. doi: 10.1093/femsec/fiac079 (PMC9341781; doi:10.1093/femsec/fiac079)
Supplement: fiac079_Supplemental_Files [file fiac079_supplemental_files.zip › S4_Supplementary_table_3.docx]

|  | Meadow | Deciduous shrub | Evergreen shrub |
| --- | --- | --- | --- |
| Deciduous shrub | org:<0.01, min:NS |  |  |
| Evergreen shrub | org:<0.01, min:<0.01 | org:NS, min:NS |  |
| Barren | org:<0.05, min:NS | org:NS, min:NS | org:NS, min:NS |

**Supplementary Table 3.** Differences in the active microbial communities between organic and mineral layers and the four different vegetation types based on pairwise PERMANOVA analysis. NS: not significant.
