# Supplementary material for: The activity and functions of soil microbial communities in the Finnish sub-Arctic vary across vegetation types
Source: FEMS Microbiol Ecol. 2022 Jul 1;98(8):fiac079. doi: 10.1093/femsec/fiac079 (PMC9341781; doi:10.1093/femsec/fiac079)
Supplement: fiac079_Supplemental_Files [file fiac079_supplemental_files.zip › S8_Supplementary_figure_5.pdf]

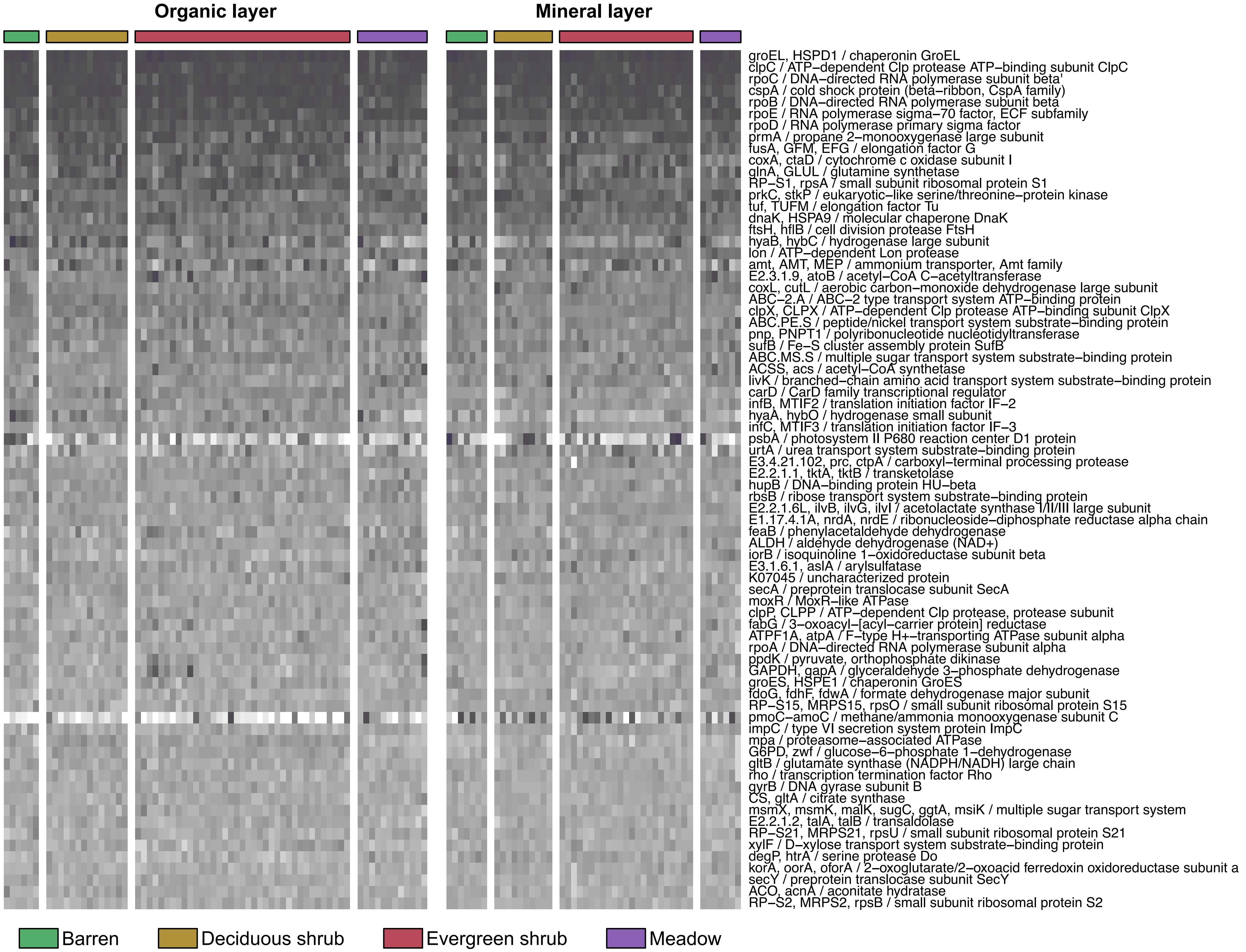

Organic layer
Mineral layer
groEL, HSPD1 / chaperonin GroEL
clpC / ATP−dependent Clp protease ATP−binding subunit ClpC
rpoC / DNA−directed RNA polymerase subunit beta'
cspA / cold shock protein (beta−ribbon, CspA family)
rpoB / DNA−directed RNA polymerase subunit beta
rpoE / RNA polymerase sigma−70 factor, ECF subfamily
rpoD / RNA polymerase primary sigma factor
prmA / propane 2−monooxygenase large subunit
fusA, GFM, EFG / elongation factor G
coxA, ctaD / cytochrome c oxidase subunit I
glnA, GLUL / glutamine synthetase
RP−S1, rpsA / small subunit ribosomal protein S1
prkC, stkP / eukaryotic−like serine/threonine−protein kinase
tuf, TUFM / elongation factor Tu
dnaK, HSPA9 / molecular chaperone DnaK
ftsH, hflB / cell division protease FtsH
hyaB, hybC / hydrogenase large subunit
lon / ATP−dependent Lon protease
amt, AMT, MEP / ammonium transporter, Amt family
E2.3.1.9, atoB / acetyl−CoA C−acetyltransferase
coxL, cutL / aerobic carbon−monoxide dehydrogenase large subunit
ABC−2.A / ABC−2 type transport system ATP−binding protein
clpX, CLPX / ATP−dependent Clp protease ATP−binding subunit ClpX
ABC.PE.S / peptide/nickel transport system substrate−binding protein
pnp, PNPT1 / polyribonucleotide nucleotidyltransferase
sufB / Fe−S cluster assembly protein SufB
ABC.MS.S / multiple sugar transport system substrate−binding protein
ACSS, acs / acetyl−CoA synthetase
livK / branched−chain amino acid transport system substrate−binding protein
carD / CarD family transcriptional regulator
infB, MTIF2 / translation initiation factor IF−2
hyaA, hybO / hydrogenase small subunit
infC, MTIF3 / translation initiation factor IF−3
psbA / photosystem II P680 reaction center D1 protein
urtA / urea transport system substrate−binding protein
E3.4.21.102, prc, ctpA / carboxyl−terminal processing protease
E2.2.1.1, tktA, tktB / transketolase
hupB / DNA−binding protein HU−beta
rbsB / ribose transport system substrate−binding protein
E2.2.1.6L, ilvB, ilvG, ilvI / acetolactate synthase I/II/III large subunit
E1.17.4.1A, nrdA, nrdE / ribonucleoside−diphosphate reductase alpha chain
feaB / phenylacetaldehyde dehydrogenase
ALDH / aldehyde dehydrogenase (NAD+)
iorB / isoquinoline 1−oxidoreductase subunit beta
E3.1.6.1, aslA / arylsulfatase
K07045 / uncharacterized protein
secA / preprotein translocase subunit SecA
moxR / MoxR−like ATPase
clpP, CLPP / ATP−dependent Clp protease, protease subunit
fabG / 3−oxoacyl−[acyl−carrier protein] reductase
ATPF1A, atpA / F−type H+−transporting ATPase subunit alpha
rpoA / DNA−directed RNA polymerase subunit alpha
ppdK / pyruvate, orthophosphate dikinase
GAPDH, gapA / glyceraldehyde 3−phosphate dehydrogenase
groES, HSPE1 / chaperonin GroES
fdoG, fdhF, fdwA / formate dehydrogenase major subunit
RP−S15, MRPS15, rpsO / small subunit ribosomal protein S15
pmoC−amoC / methane/ammonia monooxygenase subunit C
impC / type VI secretion system protein ImpC
mpa / proteasome−associated ATPase
G6PD, zwf / glucose−6−phosphate 1−dehydrogenase
gltB / glutamate synthase (NADPH/NADH) large chain
rho / transcription termination factor Rho
gyrB / DNA gyrase subunit B
CS, gltA / citrate synthase
msmX, msmK, malK, sugC, ggtA, msiK / multiple sugar transport system
E2.2.1.2, talA, talB / transaldolase
RP−S21, MRPS21, rpsU / small subunit ribosomal protein S21
xylF / D−xylose transport system substrate−binding protein
degP, htrA / serine protease Do
korA, oorA, oforA / 2−oxoglutarate/2−oxoacid ferredoxin oxidoreductase subunit a
secY / preprotein translocase subunit SecY
ACO, acnA / aconitate hydratase
RP−S2, MRPS2, rpsB / small subunit ribosomal protein S2
Barren
Deciduous shrub
Evergreen shrub
Meadow
