# Supplementary material for: The activity and functions of soil microbial communities in the Finnish sub-Arctic vary across vegetation types
Source: FEMS Microbiol Ecol. 2022 Jul 1;98(8):fiac079. doi: 10.1093/femsec/fiac079 (PMC9341781; doi:10.1093/femsec/fiac079)
Supplement: fiac079_Supplemental_Files [file fiac079_supplemental_files.zip › supplement_legends.docx]

**Supplementary table and figure legends**

**Supplementary Table S1.** Sampling sites, their coordinates and soil type sampled from (o: organic, m: mineral).

**Supplementary Table 2.** Relative abundances at the genus level in each vegetation type and soil layer.

**Supplementary Table 3.** Differences in the active microbial communities between organic and mineral layers and the four different vegetation types based on pairwise PERMANOVA analysis. NS: not significant.

**Supplementary Figure 1.** Maximum-likelihood tree of reference amino acid sequences used to discriminate between *amoA* and *pmoA* transcripts. Branch supports based on 1000 bootstraps are indicated.

**Supplementary Figure 2.** Pairwise correlation between soil physicochemical variables. Comparisons with Pearson correlation values (*r*) ≥ 0.5 or ≤ –0.5 are highlighted in red. SOM: soil organic matter; C: carbon; N: nitrogen.

**Supplementary Figure 3.** Principal coordinates analysis (PCoA) showing differences in genus-level taxonomic composition between **a)** metatranscriptomes (this study) and metagenomes (Pessi *et al*., 2021a) and **b)** soil layers in the metatranscriptomes. Ellipses in panel **b** represent 1.5 standard deviations from the group centroid.

**Supplementary Figure 4.** Heatmap showing the most abundant genes across all samples that did not match to any sequence present in the KEGG database. Abundances were square root-transformed to improve visualization.

**Supplementary Figure 5.** Heatmap showing the 100 most abundant genes that were mapped to KEGG pathways. Abundances were square root-transformed to improve visualization.

**Supplementary Figure 6.** Heatmap showing the genes belonging to metabolism pathways in KEGG. Abundances were square root-transformed to improve visualization.
