# Supplementary material for: The activity and functions of soil microbial communities in the Finnish sub-Arctic vary across vegetation types
Source: FEMS Microbiol Ecol. 2022 Jul 1;98(8):fiac079. doi: 10.1093/femsec/fiac079 (PMC9341781; doi:10.1093/femsec/fiac079)
Supplement: fiac079_Supplemental_Files [file fiac079_supplemental_files.zip › S1_Supplementary_table_1.docx]

| **site** | **point_x** | **point_y** | **org_min** |
| --- | --- | --- | --- |
| 19 | 492001.7163 | 7661066.296 | om |
| 25 | 491972.6377 | 7661365.536 | om |
| 31 | 491943.56 | 7661661.864 | om |
| 37 | 491915.594 | 7661960.17 | om |
| 91 | 492137.6413 | 7660682.24 | om |
| 97 | 492108.5998 | 7660978.766 | om |
| 103 | 492081.1879 | 7661276.182 | om |
| 109 | 492050.6532 | 7661570.414 | om |
| 115 | 492021.724 | 7661871.922 | om |
| 181 | 492189.9563 | 7661189.969 | o |
| 187 | 492159.9196 | 7661487.299 | om |
| 193 | 492133.5337 | 7661782.781 | o |
| 199 | 492102.3307 | 7662084.346 | o |
| 247 | 492360.5752 | 7660497.17 | o |
| 265 | 492273.821 | 7661395.87 | om |
| 271 | 492241.3317 | 7661690.826 | om |
| 277 | 492210.8533 | 7661988.833 | om |
| 325 | 492466.3526 | 7660411.006 | m |
| 349 | 492350.7307 | 7661605.052 | om |
| 355 | 492318.0313 | 7661898.963 | om |
| 403 | 492579.6708 | 7660322.855 | om |
| 421 | 492489.8345 | 7661217.659 | o |
| 427 | 492459.9931 | 7661515.596 | o |
| 433 | 492429.7097 | 7661815.09 | o |
| 439 | 492400.5412 | 7662109.863 | om |
| 481 | 492685.3187 | 7660233.035 | om |
| 505 | 492570.4641 | 7661423.647 | om |
| 511 | 492538.6456 | 7661720.31 | om |
| 517 | 492510.545 | 7662016.268 | om |
| 577 | 492705.3132 | 7661035.606 | om |
| 589 | 492648.6405 | 7661627.855 | om |
| 595 | 492618.6039 | 7661928.998 | om |
| 667 | 492757.2465 | 7661545.107 | om |
| 679 | 492700.207 | 7662141.065 | om |
| 733 | 492924.5712 | 7660857.792 | o |
| 739 | 492896.5419 | 7661156.949 | om |
| 745 | 492866.4088 | 7661456.958 | om |
| 751 | 492838.1235 | 7661756.274 | om |
| 757 | 492808.1934 | 7662053.996 | o |
| 811 | 493035.5804 | 7660769.903 | o |
| 823 | 492977.5755 | 7661365.71 | om |
| 835 | 492917.5722 | 7661962.542 | om |
| 889 | 493144.4042 | 7660673.747 | o |
| 895 | 493112.3677 | 7660979.071 | om |
| 901 | 493083.4854 | 7661273.049 | om |
| 913 | 493025.7016 | 7661870.94 | o |
| 919 | 492997.2759 | 7662166.759 | o |
| 961 | 493283.5397 | 7660291.576 | o |
| 967 | 493251.6385 | 7660587.25 | om |
| 979 | 493194.3672 | 7661182.349 | o |
| 1045 | 493359.8784 | 7660495.105 | o |
| 1075 | 493213.6389 | 7661987.52 | om |
| 1123 | 493470.526 | 7660406.483 | om |
| 1159 | 493295.0253 | 7662195.316 | om |
| 11201 | 492845.6659 | 7660149.446 | o |
| 11203 | 492971.6613 | 7659907.651 | o |
| 11204 | 493012.2967 | 7659781.888 | m |
| 11205 | 493103.951 | 7659750.073 | o |
| 11207 | 493267.7171 | 7659911.392 | o |
| 11208 | 493180.9779 | 7660039.125 | o |
| 11209 | 492540.0029 | 7662347.483 | o |
| 11210 | 492576.8888 | 7662442.109 | o |
| 11211 | 492578.1461 | 7662627.43 | om |
| 11212 | 492584.1153 | 7662793.33 | om |
| 11213 | 492664.3919 | 7662852.22 | om |
| 11214 | 492751.5195 | 7662776.032 | o |
| 11215 | 492751.2244 | 7662647.351 | om |
| 11216 | 492750.4017 | 7662502.435 | om |
| 11220 | 493073.521 | 7661369.919 | m |
| 11221 | 492985.5579 | 7661243.017 | om |
| 11224 | 492593.5013 | 7661897.597 | om |
| 11225 | 492854.5453 | 7661994.276 | o |

**S1 Supplementary Table S1.** Sampling sites, their coordinates and soil type sampled from (o: organic, m: mineral).
