# Supplementary figures and images for: The activity and functions of soil microbial communities in the Finnish sub-Arctic vary across vegetation types
Source: FEMS Microbiol Ecol. 2022 Jul 1;98(8):fiac079. doi: 10.1093/femsec/fiac079 (PMC9341781; doi:10.1093/femsec/fiac079)

a)

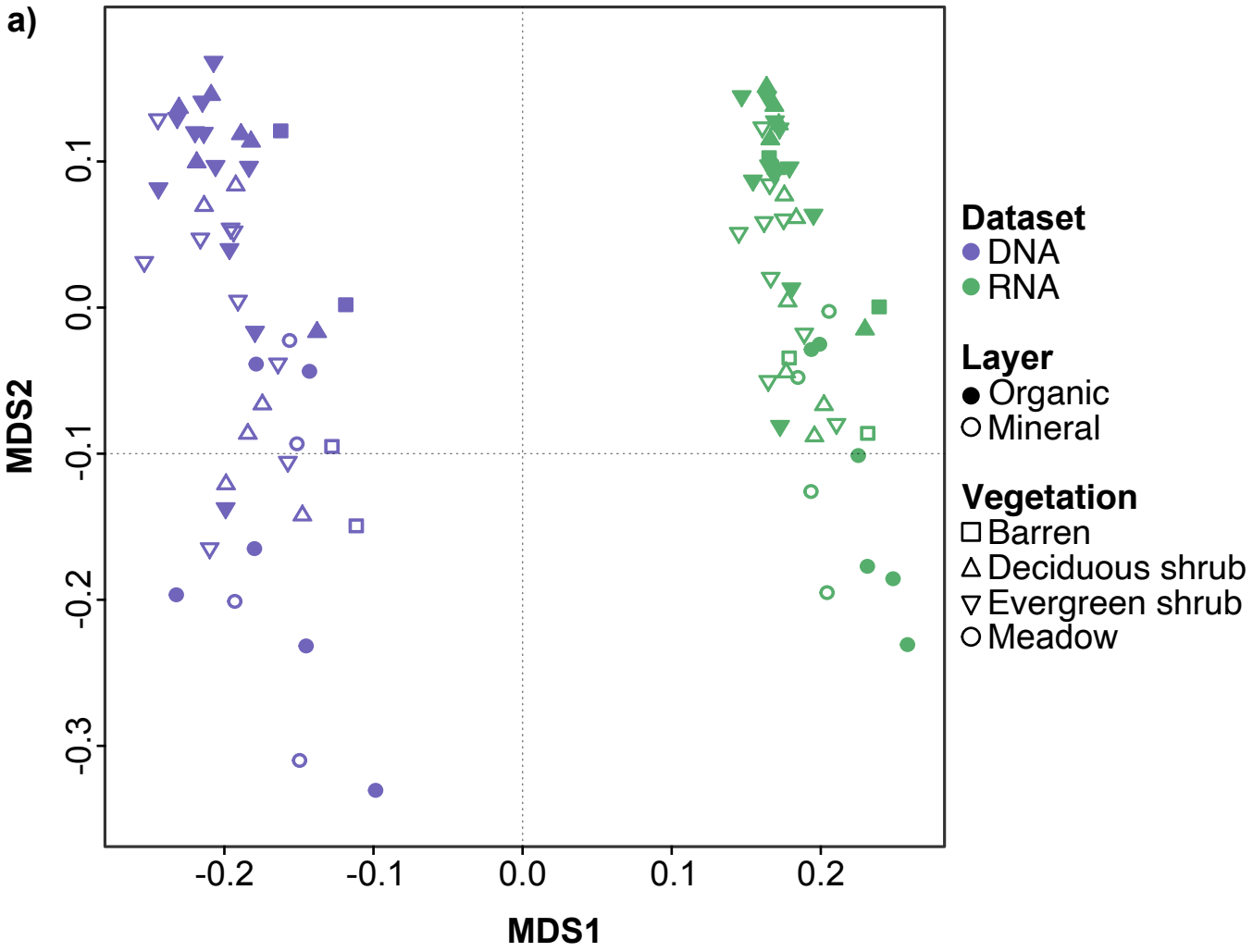

b)

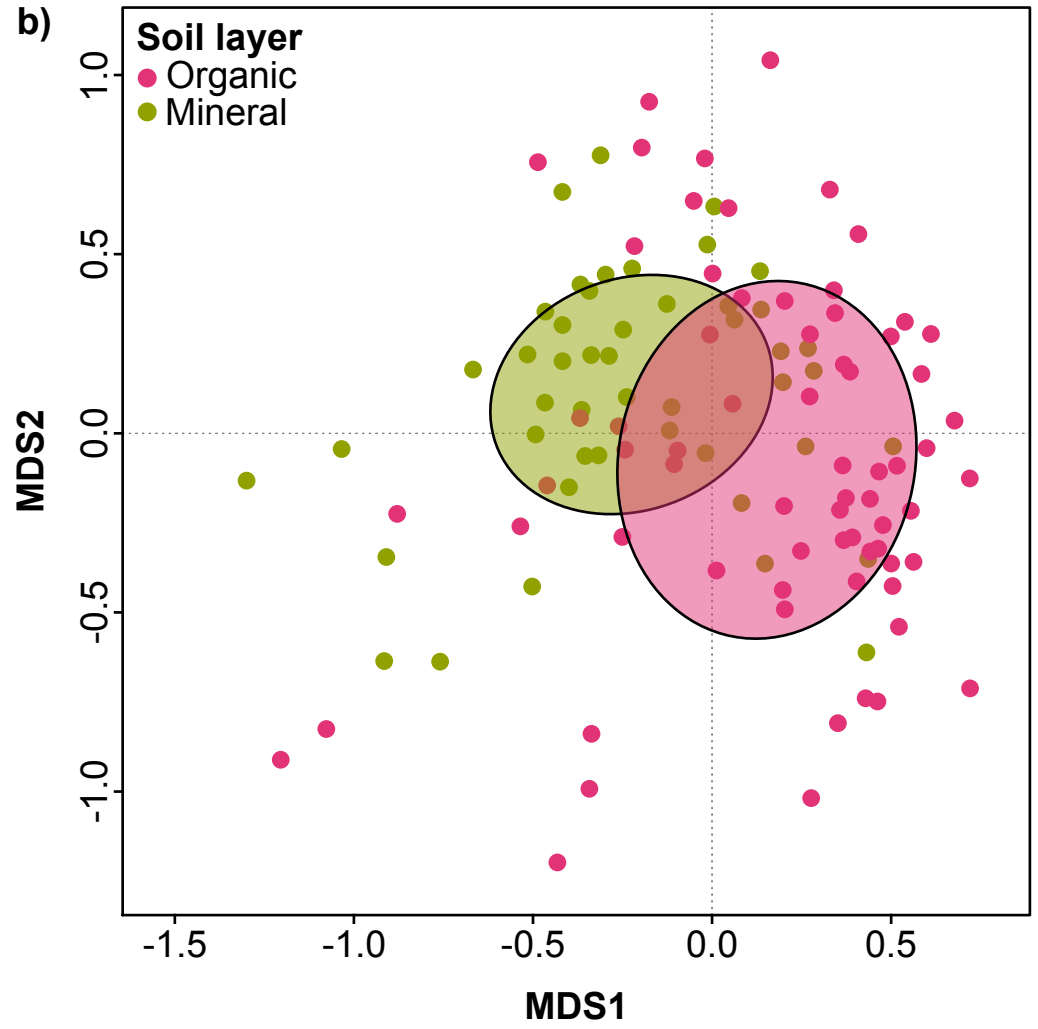

Supplement: fiac079_Supplemental_Files [file fiac079_supplemental_files.zip › S5_Supplementary_figure_3.pdf]

Organic layer

Mineral layer

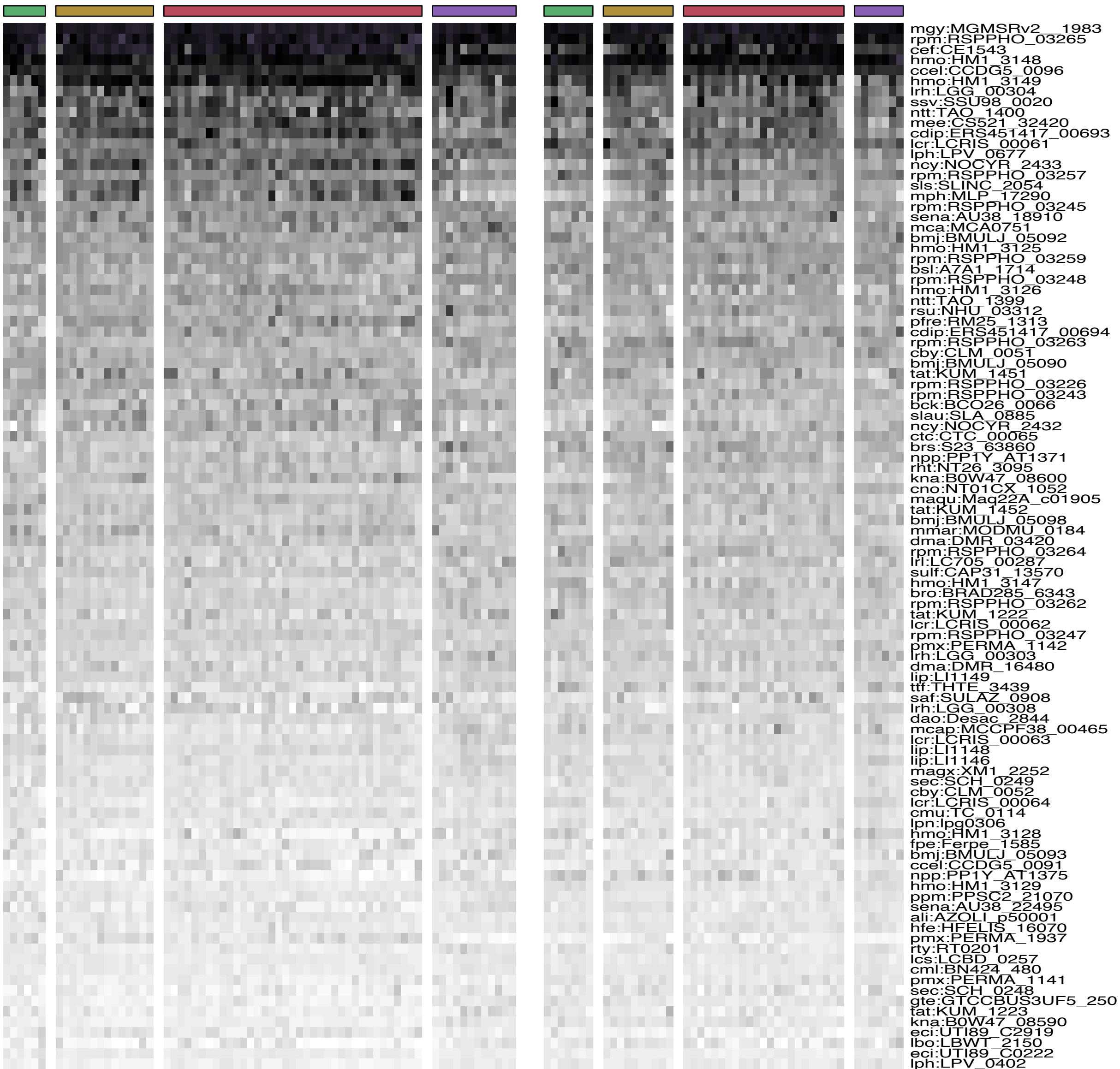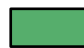

Barren

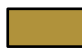

Deciduous shrub

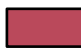

Evergreen shrub

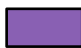

Meadow

Supplement: fiac079_Supplemental_Files [file fiac079_supplemental_files.zip › S7_Supplementary_figure_4.pdf]

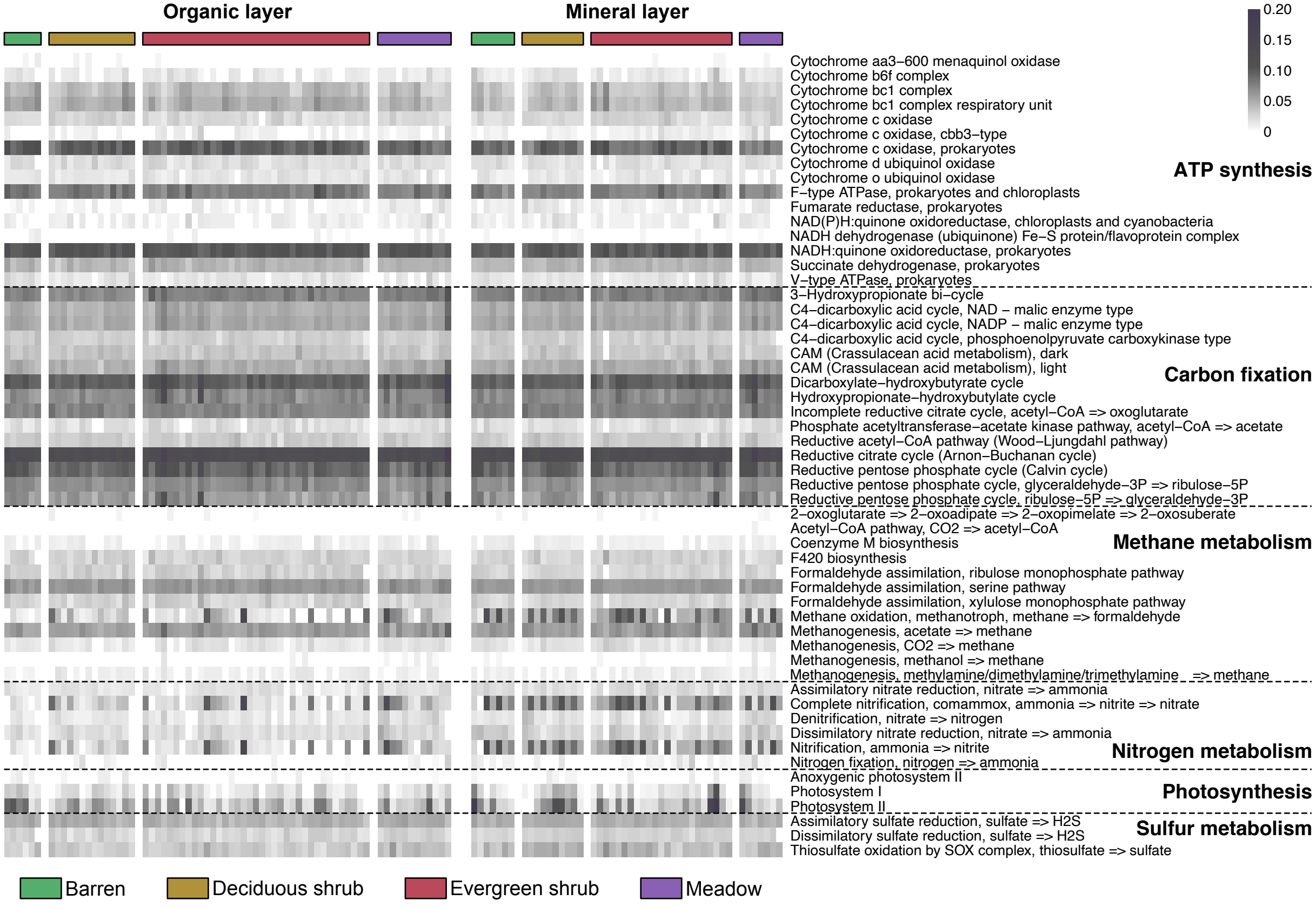

Supplement: fiac079_Supplemental_Files [file fiac079_supplemental_files.zip › S9_Supplementary_figure_6.pdf]

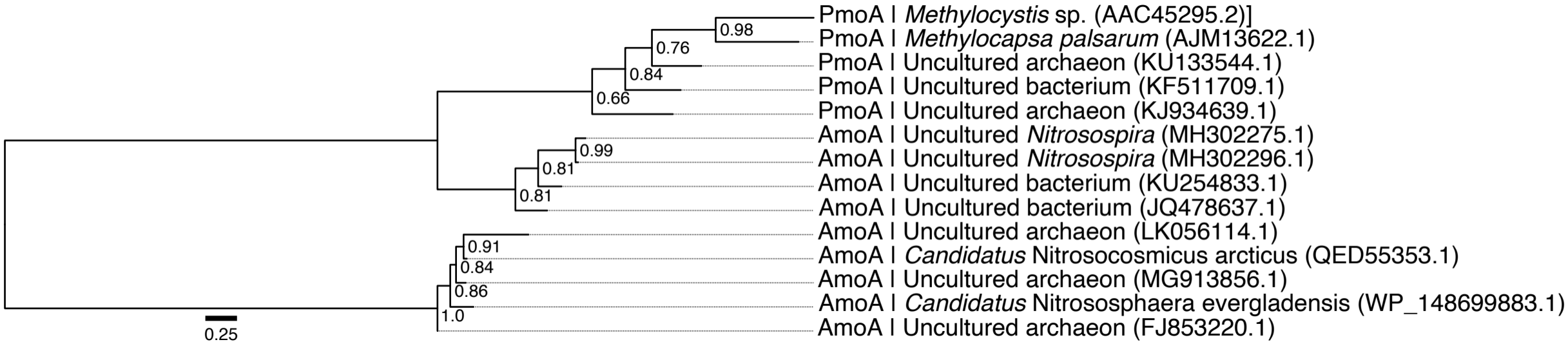

Supplement: fiac079_Supplemental_Files [file fiac079_supplemental_files.zip › S2_Supplementary_figure_1.pdf]

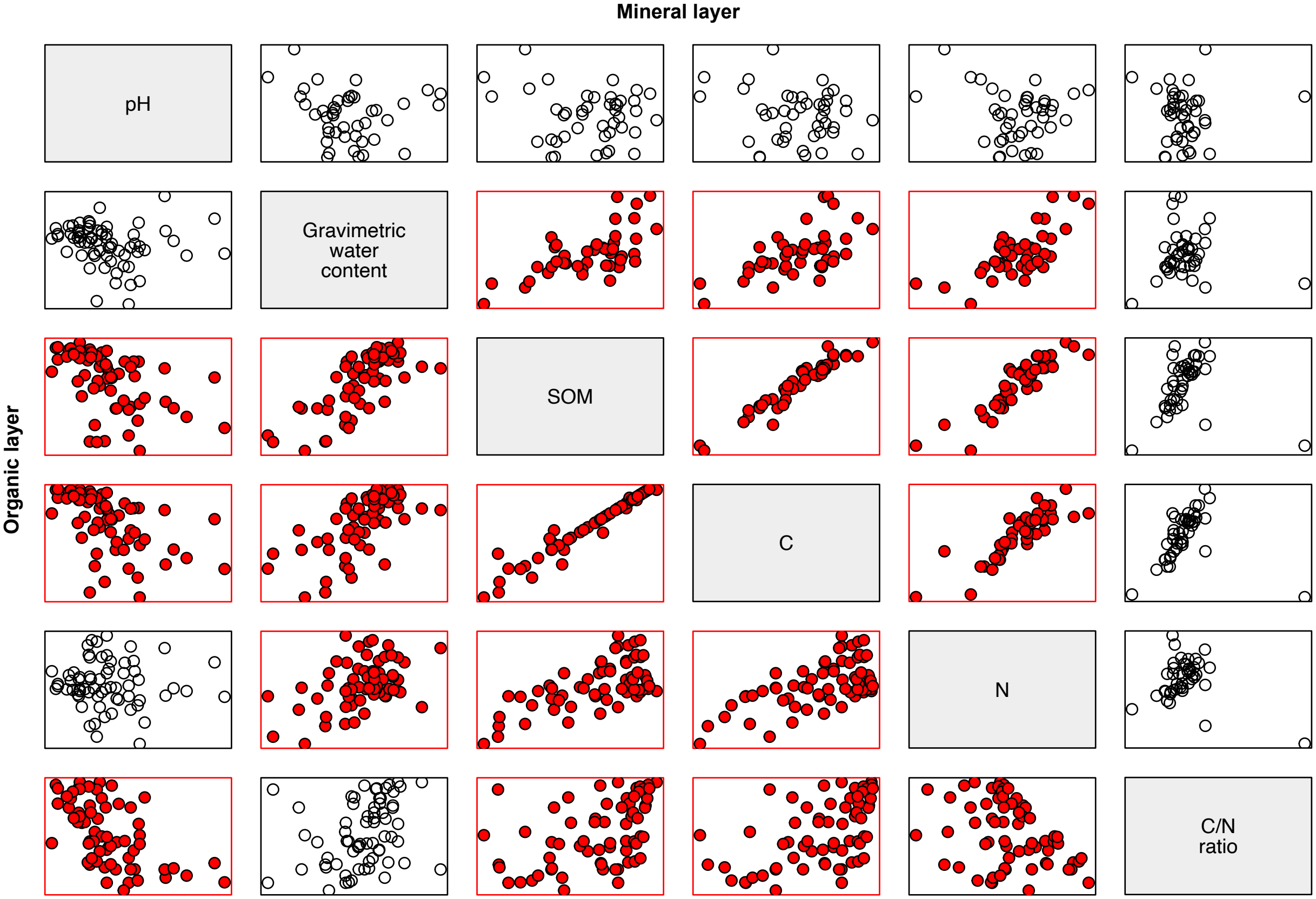

Supplement: fiac079_Supplemental_Files [file fiac079_supplemental_files.zip › S3_Supplementary_figure_2.pdf]
